# Interactive Design of Personalized Website Search Interface Based on Visual Communication

**DOI:** 10.1155/2022/2125506

**Published:** 2022-05-09

**Authors:** Zhen Xu, Shan Wang

**Affiliations:** ^1^College of Art,The Tourism College of Changchun University, Changchun 130607, Jilin, China; ^2^The Language Set, Changchun Experimental Middle School, Changchun 130117, Jilin, China

## Abstract

Aiming at the problems of low user satisfaction and long search time in the traditional interactive design method of the personalized website search interface, a personalized website search interface interactive design method based on visual communication is proposed. Under the analysis of personalized website users' search behavior, the interactive personalized website search interface is designed through a navigation module, search module, link module, interactive layout module, and visual rendering module; in the visual rendering module, the advanced texture mapping method is used to render the personalized website search interface; on the personalized website search interface, the disturbance function is imported along the normal vector, the simplified new normal vector is intelligently calculated through the concave convex texture mapping algorithm, the normal vector is solved to generate the intersection point of the high-precision interface, the illumination brightness value of each pixel of the interface is intelligently calculated, the visual communication rendering model is constructed, and the visual communication effect of the interface is improved. The simulation results show that the website interface search time of this method is within 4.9 s and the user satisfaction is up to 100%, indicating that the interaction effect of the personalized website search interface designed by this method is good.

## 1. Introduction

Since entering the 21st century, the information industry has developed rapidly. The Internet has been applied in all aspects of people's life and occupies a very important position. At present, with the rapid development of information technology, for most people, the main source of external information is browsing the web. Therefore, designing a personalized website search interface with a good user experience has become particularly important. At the same time, it has attracted more and more attention from Internet enterprises. Good user experience, easy-to-use and interesting interaction effects, and comfortable and easy-to-read visual interface are important factors to win user loyalty for the website. In people's life, the demand for Internet products is growing and the number of websites is also increasing rapidly. However, the quality of websites is also mixed [1]. In order to increase the visits of websites, attract users, and bring economic benefits, major websites have tried every means to make various distinctive personalized websites. In addition, in recent years, web technologies such as H5 and CSS3 have developed rapidly and more special effects have been applied in the design of personalized website search interface. The expression forms of personalized website search interface are more rich and diverse, which is the result of the progress of technology and the blooming of products [2]. At the same time, in the design of a personalized website search interface, some people deliberately use a large number of dynamic effects, exquisite pictures, and many technical special effects in order to excessively pursue the interface effect of the website but ignore too much in the use quality of the product itself, such as the human-computer operation interaction of the website and the friendliness of the operation experience of the website; thus, there are many so-called “vase” Internet products on the market. In this case, there will be many problems when users use the website. For example, the loading time when users open the website is too long due to the excessive use of materials on the website; when the user uses the corresponding module functions, the interactive interface is too complex or too cool, which makes the user unexpected or even surprised when operating, contrary to the user's own expectations when using the product; in addition, there are many problems, such as complex operation steps of personalized website functions, illogical use process, and so on [3]. Such problems occur when users make complaints about the use of Tucao or even quit the website, which leads to the loss of users of personalized websites [4]. This kind of problem occurs in personalized website design mainly because designers do not pay enough attention to the interface interaction design in website design when designing personalized websites, resulting in incomplete consideration in designing website products, so as to design bad websites and bring bad user experience to users; at the same time, the personalized website products designed and developed are in a dilemma and cannot be revitalized until they are eliminated by the market. Internet products are in this situation due to the failure of design. How to design a search interface with more reasonable interaction and a good experience in personalized website design is particularly important [5].

Literature [6] proposed a multisensory visual interaction interface design method based on a fuzzy median filter. Based on the description of the main contents of the multisensory visual interaction interface, the edge of the page image is extracted, the smooth image signal is obtained by calculating the smoothing function, the page image is processed by wavelet transform, and the maximum value of the page image after wavelet transform is detected. The edge points of the image are determined according to the information obtained after the wavelet transform, the gradient vector of the image is calculated, the local maximum of the gradient vector modulus is used to determine the edge of the page image, the threshold is calculated according to the noise variance and the number of pixels of the page image, and the wavelet threshold is used to denoise the page image to improve the definition of the page image; finally, the generation of multisensory visual interaction interface under unconscious behavior is realized. Document [7] proposes an interface interaction design method based on big data processing technology, constructs the database of an interface interaction system, uses the hierarchical structure design method of process constraints for interface information interaction and big data fusion, and uses the fuzzy clustering method for information clustering of interface retrieval database. The program scheduling and cross compilation of the interface are carried out under the control of the Linux kernel source code. The interface interaction design system mainly includes process management, program control, and internal file management modules. Combined with big data processing technology, the optimization design of the interface interaction system is realized. The test results show that the designed interface interaction system has good big data information processing and scheduling ability, and the recall of data is good. However, the user satisfaction with the above-given two methods is low. Document [8] proposed the design method of an HTML5 mobile interactive interface system based on Yipai 360. According to the overall framework of the Yipai 360 system hardware platform, the hardware structure is designed. According to the animation control, interactive setting, social application, and data application modules of Yipai 360pc, the mobile terminal configuration is adjusted in time through the setting of the panel and the Bluetooth/RS-485 gateway module is designed; we realize the bidirectional conversion between the signal and RS-485 signal, analyze the PLC data acquisition status in the protocol data, send the data packet to Yipai 360pc, control the digital or analog input and output according to the programmable logic controller, and complete the interface interaction design with the support of the execution and evaluation of the connection between Yipai 360 and HTML5 mobile interactive interface. Literature [9] proposed an information interface interaction design method based on unconscious cognition, analyzed unconscious cognitive behavior and information interaction efficiency by means of literature, investigation, and research, guided information interface interaction design by constructing an unconscious cognitive behavior model, and provided a theoretical basis for information interface design; information interface design based on user unconscious cognition is an important method to optimize the user experience and improve the usability and interaction efficiency of information interface, which provides a new idea and method for the study of information interface design. However, the above two methods take a long time to search the interface, resulting in low interface search efficiency.

Aiming at the problems of low user satisfaction and long search time in traditional methods, this paper proposes an interactive design method of personalized website search interface based on visual communication, uses an advanced texture mapping method to render the personalized website search interface, solves the normal vector to generate the intersection of a high-precision interface, and obtains the illumination brightness value of each pixel of the generated interface; we build a visual communication rendering model to improve the visual communication effect of the interface. Under this method, the website interface search time is within 4.9 s and the user satisfaction is up to 100%, which not only solves the problems existing in the traditional methods but also lays a foundation for improving the effect of the website user experience.

## 2. Analysis of Search Behavior of Personalized Website Users

**Figure 1:**
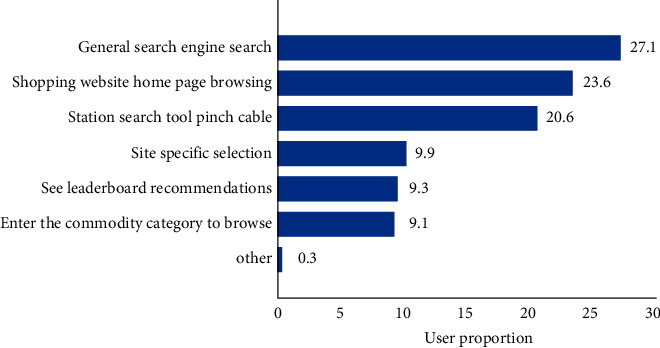
Commodity search method when Internet users shop.

Before the interactive design of the personalized website search interface, firstly, the user search behavior of a personalized website is analyzed. Users usually get the use mode of something from their daily use experience, and they will continue to use it instead of going deep into the specific principle. The great success of search engines has changed the behavior mode of users [10]. In the Internet experiment, when users are allowed to find ways to solve problems on the web page where they know at will, they will go to a search engine website in 85% of the cases. Users look for answers through search engines rather than good websites. This change in their behavior makes the website focus on building “high-viscosity” websites instead of improving the optimization of personalized websites and improving the ranking in search engines. The change in users' behavior of using personalized website search interfaces has promoted the change in personalized website function, especially the personalized website directly facing users [11]. Now, search has become an essential and important function of personalized websites. The commodity search method of Internet users when shopping is shown in Figure 1.

The proportion of users using search engines to search for goods and on-site search for goods is 27.1% and 20.6%, respectively, further highlighting the importance of search in the selection of online shopping goods [12].

**Figure 2:**
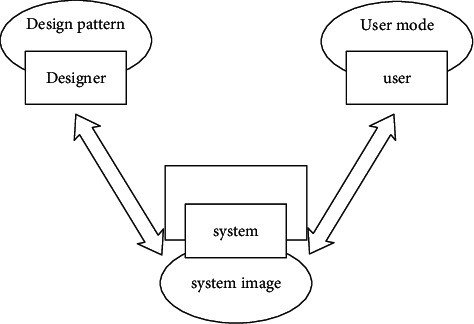
Design pattern, user pattern, and system representation.

In interface development and design, designers design products according to their own understanding of products (i.e., design psychological model), users use products according to their own understanding of products (i.e., user psychological model), and the platform for designers to communicate with users is the system. The user mode determines users' understanding of products, and the design mode determines whether product operation methods are easy to learn and use [13]. When designing products, designers must consider the user's psychological mode and design the products from the perspective that users can understand, so that the balance between the design mode and user mode is finally reflected in the product interface [14]. The design pattern, user pattern, and system representation are shown in Figure 2.

**Figure 3:**
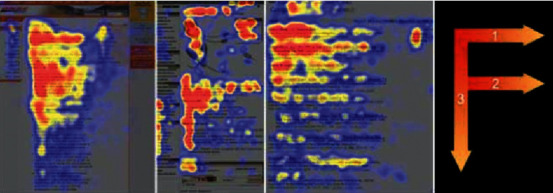
“F” browsing mode.

**Figure 4:**
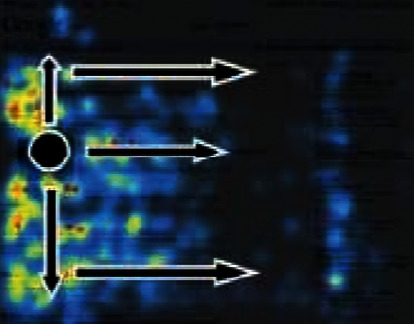
“e” browsing mode.

Users have a strong monopoly on the use of personalized websites. They will not blindly and passively accept information but actively obtain information, which also determines that users interact more autonomously and frequently when using personalized websites. It is found that most users browse the website interface with an “F” shaped path, that is, users first browse horizontally at the top of the website interface, and then the horizontal browsing distance will be shortened as the user's line of sight moves down. Finally, users quickly browse the vertical area on the left side of the interface, as shown in Figure 3 [15]. Of course, this “F” mode does not represent the browsing behavior of all users. If the information and picture content of interest to users appear below, “F” mode will also become “e,” as shown in Figure 4.

Therefore, when arranging the information priority of the website interface of colleges and universities, we can follow the visual browsing rule of users from left to right and from top to bottom and put the main information and key interaction on the upper left of the website interface, so as to meet the information needs of users in time and bring users a good information interaction experience [16].

## 3. Interactive Design of Personalized Website Search Interface Based on Visual Communication

### 3.1. Design of the Overall Architecture

**Figure 5:**
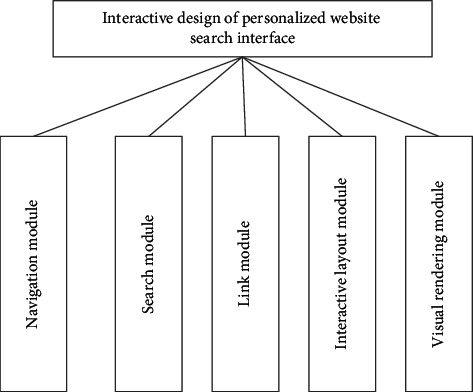
Overall design architecture.

Based on the analysis of personalized website user search behavior, this paper designs the personalized website search interface interactively through the navigation module, search module, link module, interactive layout module, and visual rendering module [17]. The overall framework is shown in Figure 5.

### 3.2. Navigation Module Design

**Figure 6:**
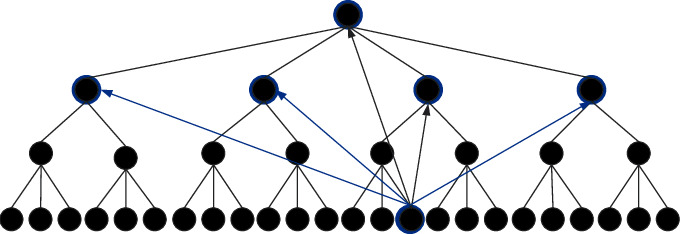
Global navigation.

Navigation is the directory of a personalized website search interface. It helps users understand their position in the personalized website and the overall structure of the website. It also guides users on where to go in the personalized website, so that users can quickly find the content and information they need. At the same time, it helps users walk freely through the site to find the content and functional elements they need. The design of navigation directly affects whether the information content of the personalized website search interface can be searched by users and browsed effectively [18]. For each kind of information, the more paths to find, the more likely it is to be read. This is also a good way to improve the browsing volume of a personalized website search interface. Therefore, the optimization of personalized website search interface navigation design can significantly promote the usability of the website. This paper uses the method of global navigation design to design personalized website search interface navigation. Global navigation mainly refers to mastering the path of the whole website and having a unified main navigation to control, as shown in Figure 6. Generally, there is global navigation on every page in the website [19]. No matter which page the user is on, accessing any other page can be realized through global navigation.

### 3.3. Search Module Design

**Figure 7:**
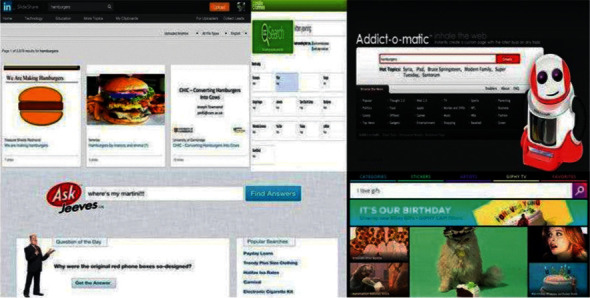
Personalized website search bar design.

The success of search engines has changed users' habits of using web pages. Search has become an important behavior of users in the website. Unless the website is really small and well organized, each page should have a search view or a link to a search page. The design of the search should be concise and follow the general formula of search: an input box, a button, and the word “search.” We should avoid using too fancy design and words. At the same time, for novice users, descriptive text can be added to the search bar to inform users of the keyword content that can be input. It is necessary to avoid indicative descriptive text similar to “input keyword” because even novice users who use the website for the first time know the function of the search bar [20]. In addition, if there is a possibility of confusing the search scope and content, you need to write it out in the search bar. The design of a personalized website search bar is shown in Figure 7.

### 3.4. Link Module Design

The interaction between users and personalized websites is mainly completed through links. According to the user's usage habits, the buttons and links that can be clicked at the obvious signs can improve the usability of the web page. The buttons in the web page are clickable, which is obtained from the user's experience [21]. Therefore, the marking of text links is a place to pay attention to in the principle of ease of use. For the navigation of personalized websites or all links in websites, you can remove the underline design and just change the color or add an underline when the mouse moves to the link text. For the mixed arrangement of ordinary text and hyperlink text, it is necessary to clearly distinguish the difference between the hypertext link and ordinary text [22]. In addition, you can design text links as button icons.

### 3.5. Interactive Layout Module Design

As an expressive visual language, personalized website search interface design pays special attention to the interactive layout of the interface. The layout of the web interface directly affects the convenience of users using the interface information. A reasonable interactive layout will enable users to quickly find the core content and services. On the contrary, they do not know how to obtain the required information, or how to browse to get the corresponding service, and then the user will choose to leave [23].

**Figure 8:**
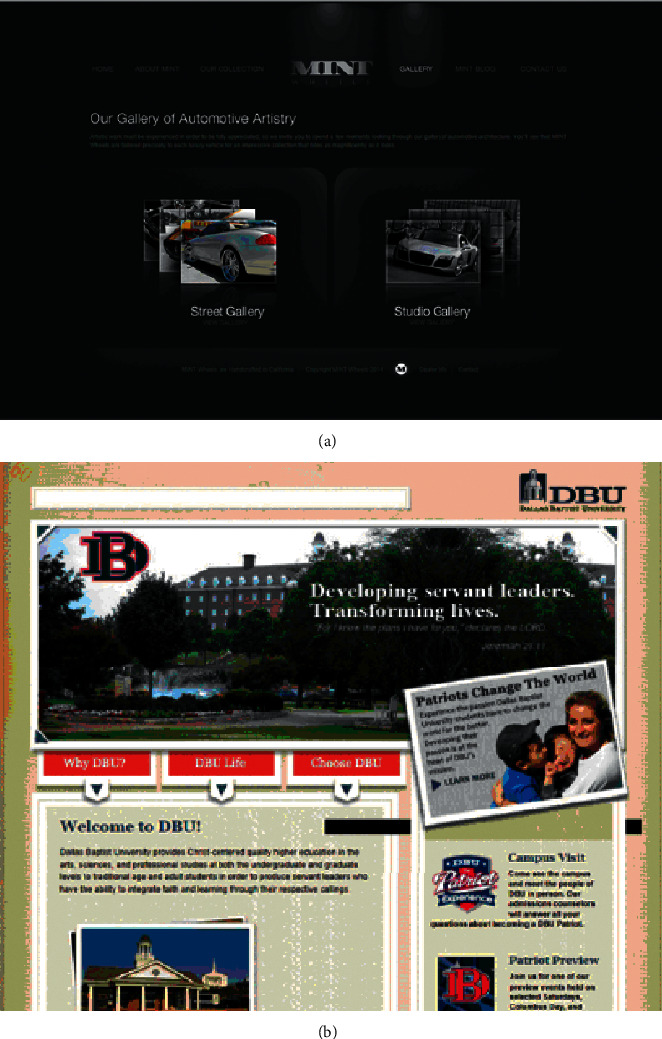
Interface layout of (a) Mint Wheels website and (b) Dallas Baptist University website.

Although the personalized website search interface layout does not attract the user's visual attention to a certain location or object as other elements such as color and graphics, a good interface layout often becomes a prerequisite for attracting the user's attention and trying to choose and the user does not tend to be in a chaotic state. It takes time and energy to pay attention to an element or content in a layout without a sense of stability. Therefore, a good interactive layout first ensures that the user's visual attention is stimulated. To define whether an interactive layout is chaotic, it is first necessary to ensure that the user's visual weight on the layout reaches a certain balance; when the elements in the interface are gathered together, the visual weight is formed. The visual weight is virtual and obtained through the user's visual perception. Generally speaking, in order to achieve the balance of the interface layout, these visual weights must be offset by a weight with equal and opposite weight; otherwise, the layout will show an unstable state. A balanced layout can make the shift of sight operate in an orderly manner within a reasonable range without psychological burden and pressure [24]. A balanced layout design makes users feel stable and simple, which is often more attractive. The interface layout between the Mint Wheels website and the Dallas Baptist University website is shown in Figure 8.

As shown in Figure 8, in the Mint Wheels web interface, the logo is placed on the central axis of the interface and on the top to help establish a symmetrical balance. Moreover, the visual weight on both sides of the central axis is the same and the elements on both sides also form a corresponding relationship [25]. This makes the web interface layout delicate and concise and complements its content. However, such a symmetrical visual weight balance will inevitably appear rigid and lifeless, so the concept of balance cannot be achieved only by symmetry. When the elements on both sides are asymmetric, they can also achieve the balance of visual weight. As shown in Figure 8, the Dallas Baptist University web interface is particularly asymmetric compared with the Mint Wheels web interface, but visually, the logo balances the search box, the loose large content area on the right side of the interface balances the tight small content area on the right side, but the asymmetric arrangement of elements achieves the balance of weight, which will greatly enhance the possibility of users' visual selective attention [26].

### 3.6. Design of the Visual Rendering Module

The personalized website search interface includes two elements: graphics and text, and these two visual elements have one thing in common, that is, these two elements are the media to convey page information to users through visual color [27]. Color is a highly stimulating and powerful design element. Color can convey a kind of information to the browser of the interface, so as to reflect the user's psychological feelings and stimulate the user's psychological activities. Therefore, color is often easier to attract the user's attention and attract the user's attention than other elements.

Because the perception of the light wave by human eyes has a length range and different colors have different light wave lengths, people's perceptions of different colors will be different. In general, the length of human perception of light wave ranges from 400 to 700 *μ*m; when the color wavelength is perceived by the human eye at both ends of the light wave range, i.e., 400 *μ*m (red) or 700 *μ*m (purple), the brightness of the color will weaken. Too strong or too weak brightness of the color will cause people's visual fatigue, so when selecting the color, one must try to choose the light wave length suitable for people's vision to create a comfortable interactive environment. If people look at a position for a long time, they will feel that their sight is becoming more and more blurred. This phenomenon is called visual residue [28]. Therefore, in order to improve the visual quality of the personalized website search interface and improve the visual communication effect of the interface, when designing the personalized website search interface, the personalized website search interface is rendered through the advanced texture mapping method, which includes concave-convex and normal texture mapping algorithms:

The flow of the bump texture mapping algorithm is as follows.

**Step 1 .**

Suppose that the web page crawling function *P* of the personalized website search interface is as follows:

(1) $$\begin{array}{c} P=P\left(x,y\right), \end{array}$$

where (*x*, *y*) represents texture coordinates [29].

**Step 2 .**

Calculate the partial derivative of formula (1) to obtain *P*_*x*_ and *P*_*y*_; then, the normal vector *E* of point 1 on the personalized website search interface is shown in the following formula:

(2) $$\begin{array}{c} E=\frac{\left(P_{x}\times P_{y}\right)}{\left|P_{x}\times P_{y}\right|}. \end{array}$$

**Step 3 .**

On the personalized website search interface, each point imports a small disturbance function along the normal vector, as shown in the following formula:

(3) $$\begin{array}{c} P^{\prime }=P\left(x,y\right)+S\left(x,y\right)\cdot E. \end{array}$$

Here, the surface equation of the new personalized website search interface is described by *P*′ and the disturbance function is described by *S*(*x*, *y*).

Calculate the partial derivative of *P*′ in *x*, *y* direction, as shown in the following formulas:

(4) $$\begin{array}{c} P_{x}^{\prime }=P_{x}^{\prime }+S_{x}\cdot E+S\left(x,y\right)\cdot E_{x}, \end{array}$$

(5) $$\begin{array}{c} P_{y}^{\prime }=P_{y}+S_{y}\cdot E+S\left(x,y\right)\cdot E_{y}. \end{array}$$

After simplification, the new normal vector of each point in the personalized website search interface is obtained, as shown in the following formula:

(6) $$\begin{array}{c} E^{\prime }=P_{X}^{\prime }\times P_{W}^{\prime }=P_{X}\times P_{Y}+S_{X}\left(E\times P_{Y}\right)+S_{Y}\left(P_{X}\times E\right)\\ =E+S_{X}\left(E\times P_{Y}\right)+S_{Y}\left(P_{X}\times E\right), \end{array}$$

where the disturbance factor is described by *S*_*X*_(*E* × *P*_*Y*_) and *S*_*X*_(*P*_*Y*_ × *E*). Under the action of light, the surface of a personalized website search interface will produce an uneven rendering effect [30].

**Figure 9:**
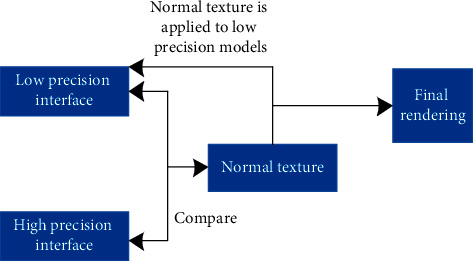
Normal texture mapping structure.

In order to improve the effect of the bump texture mapping algorithm, it is improved by normal texture mapping, as shown in Figure 9.

As can be seen from Figure 9, in order to solve the normal vector, emit rays from the low to high interface, generate the intersection of a high-precision interface, generate the illumination brightness value of each pixel of the interface, and construct the visual communication rendering model. The expression is

(7) $$\begin{array}{c} FB=E\left(P_{X}^{\prime }\times P_{W}^{\prime }\right)P\left(x,y\right). \end{array}$$

Therefore, the rendering effect of the personalized website search interface is improved.

## 4. Simulation Experiment Analysis

### 4.1. Experimental Design

In order to verify the effectiveness of the interactive design method of personalized website search interface based on visual communication in practical application, a simulation experiment is carried out. The experiment adopts the general configuration of the current mainstream PC, and the compilation and running environment adopt common tools. Specific parameters are shown in Table 1.

**Figure 10:**
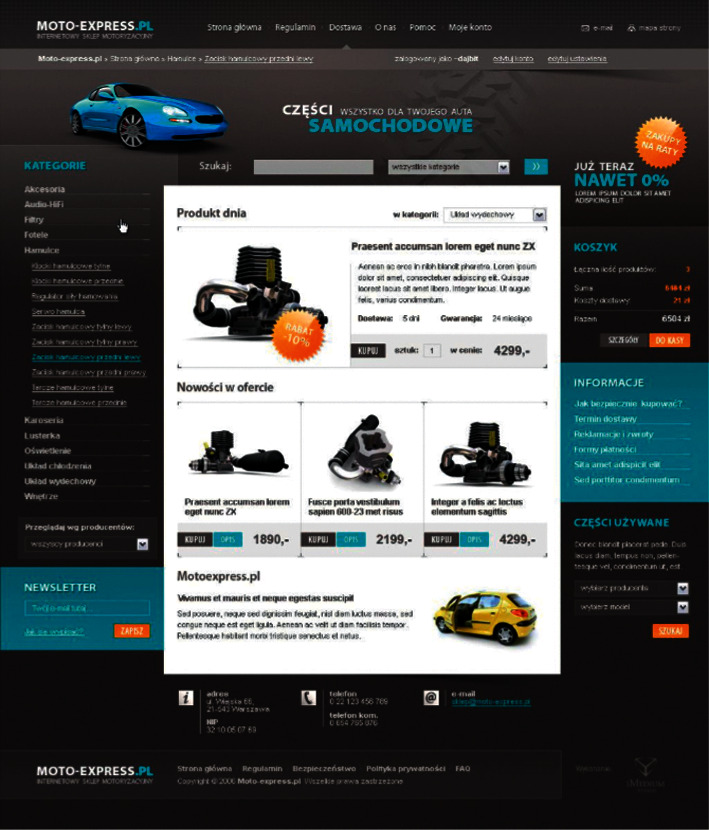
Experimental object.

Under the above-given experimental environment, this paper selects an automobile website as the experimental object for the experimental test. The experimental object is shown in Figure 10.

### 4.2. Experimental Analysis

#### 4.2.1. Website Interface Search Time

The interactive design method of the personalized website search interface based on visual communication proposed in this paper, the multisensory visual interactive interface design method based on the fuzzy median filter proposed in the literature [6], and the interface interactive design method based on big data processing technology proposed in interface literature [7] are used to search the automobile website interface and test the time consumed by the three search methods. The test results are shown in Table 2.

According to Table 2, when the number of experiments is 50, the website interface search time of the method in [6] is 11.7 s, the website interface search time of the method in [7] is 18.6 s, and the website interface search time of our method is 3.7 s; when the number of experiments is 100, the website interface search time of the method in [6] is 14.5 s, the website interface search time of the method in [7] is 24.6 s, and the website interface search time of our method is 4.9 s; the interactive design method of the personalized website search interface based on visual communication proposed in this paper takes 4.9 s to search the personalized website interface, which is shorter than the multisensory visual interactive interface design method based on fuzzy median filter proposed in the literature [6] and the interface interactive design method based on big data processing technology proposed in the literature [7]. This is because this method solves the normal vector by emitting rays from the low to high interface, generates the intersection point of the high-precision interface, obtains the illumination brightness value of each pixel of the generated interface, constructs a visual communication rendering model, improves the rendering efficiency of the personalized website search interface, and effectively improves the website interface search efficiency of this method.

#### 4.2.2. User Satisfaction

**Figure 11:**
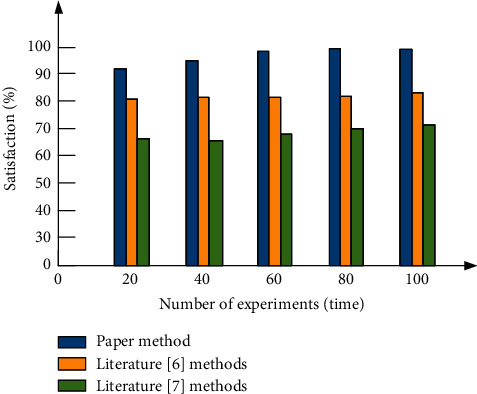
Comparison results of user satisfaction.

In order to further verify the effectiveness of this method, the personalized website search interface interactive design method based on visual communication proposed in this paper, the multisensory visual interactive interface design method based on fuzzy median filter proposed in [6], and the interface interactive design method based on large data processing technology proposed in [7] are used for personalized website interface search. The satisfaction of users is tested, and the test results are shown in Figure 11.

According to Figure 11, when the number of experiments is 20, the user satisfaction of the method in [6] is 80%, the user satisfaction of the method in [7] is 68%, and the user satisfaction of the method in this paper is as high as 92%; when the number of experiments is 60, the user satisfaction of the method in [6] is 81%, that of the method in [7] is 69%, and that of our method is as high as 99%; after applying the interactive design method of personalized website search interface based on visual communication proposed in this paper, the user satisfaction is up to 100%, which shows that the interactive effect of personalized website search interface designed by our method is good. This is because this method uses the advanced texture mapping method to render the personalized website search interface, improve the visual communication effect of the interface, and improve the user satisfaction.

## 5. Conclusion

This paper proposes an interactive design method of a personalized website search interface based on visual communication and uses an advanced texture mapping method to render the personalized website search interface; through the concave-convex texture mapping algorithm, we intelligently calculate the simplified new normal vector, solve the normal vector to generate the intersection of the high-precision interface, intelligently calculate and generate the illumination brightness value of each pixel of the interface, build the visual communication rendering model, and improve the visual communication effect of the interface. The following conclusions are drawn through experiments:

1. The interactive design method of personalized website search interface based on visual communication proposed in this paper takes 4.9 s to search the personalized website interface, and the personalized website interface search time of this method is short
2. When the number of experiments is 60, the user satisfaction of this method is as high as 99%; after applying the interactive design method of personalized website search interface based on visual communication proposed in this paper, the user satisfaction is up to 100%, which shows that the interactive effect of personalized website search interface designed by this method is good

This paper has achieved high user satisfaction, but the search time still needs to be improved, and further research is needed in the future.

## Figures and Tables

**Table 1:** Experimental environment parameters.

Serial number	Name	Parameters
1	Central processing unit	Intel (R) Core (TM) 2 Duo CPU E7500@2.93 GHz 2.94 GHz
2	Installed memory	8.00 G
3	Display adapter	NVIDIA GeForce GTX 550 Ti
4	Development tool	Visual Studio 2010
5	Language and corresponding library	C/C++, OpenCV 2.4.3, Qt4.7.4, Matlab R20I3a
6	System environment	Windows 7

**Table 2:** Website interface search time of three methods.

Number of experiments/time	This paper's method	Method in literature [6]	Method in literature [7]
10	2.6	10.6	15.6
20	2.9	10.7	17.2
30	3.1	10.8	18.6
40	3.5	11.5	18.2
50	3.7	11.7	18.6
60	3.9	11.9	19.2
70	4.1	12.5	19.8
80	4.5	12.8	20.1
90	4.6	13.2	23.5
100	4.9	14.5	24.6

## Data Availability

The dataset can be accessed upon request.
